# Large Lemurs: Ecological, Demographic and Environmental Risk Factors for Weight Gain in Captivity

**DOI:** 10.3390/ani10081443

**Published:** 2020-08-18

**Authors:** Emma L. Mellor, Innes C. Cuthill, Christoph Schwitzer, Georgia J. Mason, Michael Mendl

**Affiliations:** 1 Bristol Veterinary School, University of Bristol, Langford House, Langford, Bristol BS40 5DU, UK; mike.mendl@bristol.ac.uk; 2 School of Biological Sciences, University of Bristol, Life Sciences Building, 24 Tyndall Avenue, Bristol BS8 1TQ, UK; i.cuthill@bristol.ac.uk; 3 Dublin Zoo, Phoenix Park, Dublin 8, D08 WF88, Ireland; christoph.schwitzer@dublinzoo.ie; 4 Department of Animal Biosciences, University of Guelph, 50 Stone Road East, Guelph, ON N1G 2W1, Canada; gmason@uoguelph.ca

**Keywords:** obesity, body mass, fat storage, captive primates, animal welfare, zoo animals

## Abstract

**Simple Summary:**

Excessive body mass, i.e., being overweight or obese, is a health concern. Some lemur species are prone to extreme weight gain in captivity, yet for others a healthy body condition is typical. The first aim of our study was to examine possible ecological explanations for these species’ differences in susceptibility to captive weight gain across 13 lemur species. Our second aim was to explore demographic and environmental risk factors across individuals from the four best-sampled species. We found a potential ecological explanation for susceptibility to captive weight gain: being adapted to unpredictable wild food resources. Additionally, we also revealed one environmental and four demographic risk factors, e.g., increasing age and, for males, being housed with only fixed climbing structures. Our results indicate targeted practical ways to help address weight issues in affected animals, e.g., by highlighting at-risk species for whom extra care should be taken when designing diets; and by providing a mixture of flexible and fixed climbing structures within enclosures.

**Abstract:**

Excessive body mass, i.e., being overweight or obese, is a health concern associated with issues such as reduced fertility and lifespan. Some lemur species are prone to extreme weight gain in captivity, yet others are not. To better understand species- and individual-level effects on susceptibility to captive weight gain, we use two complementary methods: phylogenetic comparative methods to examine ecological explanations for susceptibility to weight gain across species, and epidemiological approaches to examine demographic and environment effects within species. Data on body masses and living conditions were collected using a survey, yielding useable data on 675 lemurs representing 13 species from 96 collections worldwide. Data on species-typical wild ecology for comparative analyses came from published literature and climate databases. We uncovered one potential ecological risk factor: species adapted to greater wild food resource unpredictability tended to be more prone to weight gain. Our epidemiological analyses on the four best-sampled species revealed four demographic and one environmental risk factors, e.g., for males, being housed with only fixed climbing structures. We make practical recommendations to help address weight concerns, and describe future research including ways to validate the proxy we used to infer body condition.

## 1. Introduction

Excessive body mass, i.e., being overweight or obese, is a health concern. Such conditions are associated with fat-levels that may impair health [1], and problems such as diabetes, heart disease, impaired reproduction, orthopaedic disorders, and cancers [2,3,4,5,6,7]. Methods of determining body condition include weight-for-height (kg/m^2^) indices in humans (“body mass index”: 1) and other primates (e.g., [8]), skinfold thickness (e.g., [9]), and visual body condition scoring systems (e.g., [10,11,12]). Body mass is also used as a proxy for body fat: individuals ≥20–25% over the ideal are considered obese (e.g., in humans, dogs, *Canis lupus familaris*, and cats, *Felis catus*: [7,13]). Positive energy balance, i.e., calorific intake greater than expenditure, is central to weight gain [13,14] but is not the sole factor. Energy balance is affected by environmental, e.g., diet and physical activity levels, and genetic factors [13], e.g., obesity-risk varies between human [15] and non-human animal populations [6,14,16], and is heritable (e.g., vervet monkeys, *Chlorocebus pygerythrus*: [17]).

Lemuriformes (“lemurs”) are a primate infraorder native to Madagascar, and these species vary in susceptibility to weight gain in captivity. For instance, a healthy body condition is typical of greater bamboo, *Prolemur simus*, and red-bellied lemurs, *Eulemur rubriventer* [18,19]. Conversely, ring-tailed, *Lemur catta*, and blue-eyed black lemurs, *E. flavifrons*, are prone to elevated body masses [18,19], and for some species problems such as obesity are prevalent. For example, using a weight-based method of inferring body condition, 46.5% black-and-white ruffed and red ruffed lemurs, *Varecia variegata* and *V. rubra*, housed in European zoos were deemed obese (defined by the authors as ≥two standard deviations from the wild mean weight: [20]). Similarly, of 902 zoo lemurs from 14 species 54% were considered overweight or obese (likewise defined as ≥ two and ≥ four standard deviations from species-typical wild mean weights, respectively: [18]). Furthermore, obesity can hinder breeding programmes, and obese animals are unsuitable for wild reintroduction [21]—concerning for a taxonomic group as threatened as lemurs (cf. [22]). Therefore, to improve health and promote conservation, understanding the basis for some species’ susceptibility to weight gain is fundamental.

Three aspects of species-typical ecology might explain species differences in susceptibility to weight gain. The first relates to lemurs’ physiological and behavioural adaptations regarding fat-storage [23], to buffer against future resource-restriction (i.e., “thrifty genotypes” sensu: [24]). Madagascar has relatively poor soil quality and plant productivity, with a harsh and unpredictable climate, the severity of which varies geographically [25]. Rainfall affects plant growth [26,27] and annual rainfall is commonly used to infer primary productivity and thus food availability [28,29,30]. Annual rainfall varies across Madagascar, e.g., northern areas have a very long dry season and the south receives very little rainfall, unlike eastern areas [31,32,33]. Additionally, there are regional differences in between-year annual rainfall, e.g., the north and south are especially variable; hypothesised to explain unpredictable fruit availability [34]. The frequency and severity of droughts, cyclones and El Niño events also vary across the island and between years—events which also restrict food resources [25,35,36,37]. Therefore, while all lemur species naturally experience relatively poor and unpredictable food resources, this will be more severe for some than others, according to where they reside on the island. Additionally, captive and wild food quality likely differ. Commercial fruits especially are more energy- and sugar-rich than wild equivalents—a mismatch cited in the excessive body masses observed in some captive lemurs (e.g., [38,39]). In captivity, this mismatch combined with thrifty adaptations to naturally poor and/or unpredictable food resources might leave certain species susceptible to weight gain.

The second aspect of species-typical ecology that might explain species variation in captive weight gain, is arboreality. Ring-tailed lemurs, for instance, are deemed “semi-terrestrial” [40], aye ayes, *Daubentonia madagascariensis*, also spend time at ground-level [41], and grey mouse lemurs, *Microcebus murinus*, hunt and occasionally nest on the ground [42]. However, other species are arboreal specialists, such as ruffed lemurs [43], only coming to ground if absolutely required, e.g., to drink [44] or to retrieve fallen young [45]. Across mammals, including lemurs, terrestrial species carry higher fat stores than arboreal ones [46]. Arboreality imposes upper limits on how much body fat species develop, as carrying excess weight imposes locomotive and agility costs [46,47,48,49]. Therefore, being naturally arboreal could be *protective* against captive weight gain, meaning such species should be less prone to excessive body masses than those more terrestrial.

Finally, predation risk could drive species differences in susceptibility to weight gain. High predation pressure is associated with relatively smaller fat stores in birds, reptiles and mammals, because carrying excess weight impedes escape [48,49,50,51,52,53,54]. To the best of our knowledge, there is no evidence for such patterns in lemurs. However, lifespan in wild grey mouse lemurs was negatively correlated with body mass, suggested to result from selective predation on larger individuals [55]. High wild predation risk, therefore, could explain why some species are less prone to weight gain even when in captivity.

In addition to species-typical ecology, an individual’s demographic profile and environment, e.g., diet and physical activity, also affect body condition (e.g., [13]). Epidemiological approaches [56] are useful in exploring such individual-level effects, having been successfully used to examine risk factors for health and welfare problems across zoo [57], farm [58], and companion animals [59,60]. For captive lemurs, usable space and exercise opportunities (sensu [13]), the need to maintain body temperature if housed outdoors (sensu [61]), use of contraceptives/neutering [14,19,62], amount of fruit in the diet (cf. [38]), and activity levels (sensu [61]) are biologically relevant aspects that could contribute to susceptibility to weight gain.

Here, we use two complementary methods (cf. [63]) to better understand susceptibility to weight gain in captive lemurs. We use phylogenetic comparative methods to test the hypotheses relating species-typical ecology to captive weight gain described above, aiming to identify *species*-level effects, i.e., ecological risk factors. We also use an epidemiological approach, and by comparing across individuals we aim to identify *individual*-level effects, e.g., demographic and/or environmental risk factors for captive weight gain.

## 2. Materials and Methods

Ethical approval for outcome data collection was granted by the Faculty of Health Sciences Research Ethics Committee of the University of Bristol (University Investigation Number: 37201).

### 2.1. Outcome Variable

As reviewed above, there are several ways to determine body condition in primates, but most require direct access and/or handling of animals (e.g., waist circumference measurements: [64]), and none to-date have been tested and validated for use in lemurs. A practical alternative is to use deviations from a species’ normal mass to infer body condition. Many zoos routinely weigh their animals [65], and published values of lemur species-typical mean wild adult weights are available [66]. Comparisons between species-typical wild mean and captive representatives’ body masses have already been used to infer body condition across several lemur species [18], and similar weight-based methods of inferring body condition are used in other species (e.g., in humans, dogs, and cats [7,13,67]. We acknowledge, however, that using mass alone to infer body condition is limited because frame is not accounted for (unlike, for example, body mass index: [1]); however, frame measurements are rarely readily available for captive wild animals.

Here, we use “relative body mass” as a proxy for body condition, i.e., the ratio between a captive animal’s body mass and its species-typical wild mean (method after: [18], wild means from: [66]). Relative body mass values correspond with the following body conditions (after [18]): <0.75 = *underweight*, i.e., the captive animal is <75% of its wild mean; 0.75–1.25 = *healthy,* the captive animal is 75–125% of its wild mean; 1.25–1.5 = *overweight,* the captive animal is 125–150% of its wild mean; 1.5–2 = *obese*, the captive animal is 150–200% of its wild mean; and ≥2 = *morbidly obese*, the animal is 200% (or more) of its wild mean.

Our outcome for comparative analyses was species-typical median relative body mass, calculated from values from individuals across 13 species (medians being the preferred species summary statistic to reduce the effects of outliers and skew in the raw data: [68]). For epidemiological analyses of the four best-sampled species, our outcome was individuals’ relative body masses.

### 2.2. Outcome Data Collection and Processing

Data on body masses and corresponding living conditions of captive lemurs were collected between August 2016–January 2018 mainly using an online survey made in Google Forms (see Supplementary Materials File S1 for the questions). Participants could either provide body mass values within the survey or provide Zoological Information Systems (ZIMS) Specimen Reports for their lemurs. As the online survey was suitable for collections with ≤10 enclosures, larger collections completed a tailored Excel spreadsheet containing the same questions. On request, the Duke Lemur Center (www.lemur.duke.edu) kindly provided detailed copies of their most recent records of body masses (an update of the dataset of [69]), and housing, feeding and enrichment routines of their lemurs.

Using ‘species holding’ information from Species360 (then, the International Species Information System [70]) we emailed or used zoo website contact forms to recruit zoos known to hold lemurs. After initial contact, up to two further reminder requests were made. Zoos were classed as non-participatory if no response was received after the third email, or if the zoo declined to participate. The British and Irish Association of Zoos and Aquariums (BIAZA) Research Committee, the Association of Zoos and Aquariums (AZA, North America) Prosimian Taxon Advisory Group (TAG), and the Zoo and Aquarium Association (ZAA, Australasia) Primate TAG all supported our project.

Raw online survey responses were downloaded for processing, and responses contained in tailored Excel spreadsheets and the Duke Lemur Center’s data were added to this, resulting in records from 1386 animals representing 22 species from 133 collections worldwide. Initially, we processed these raw data into individual-level responses, combining responses from specific questions where necessary to calculate our variables of interest, ready for calculation of species summary statistics for the comparative analyses (see Table 1). A subset of these individual-level responses from the four best-sampled species (Section 2.2.2) were used for epidemiological analyses. Note that the resulting datasets contain missing data for some variables resulting in sample size differences.

We excluded entries without body mass values, those from species-hybrids (because we were interested in species effects), and females known to be pregnant (as their weights might over-estimate their body condition). We calculated age at weighing from ZIMS Specimen reports or reported dates of birth to determine which lemurs were adult; for the minority of animals lacking this information, we accepted the respondents’ judgement of “adult”. Lemurs have a late “near-adult” growth period, in which animals are sexually mature but not yet fully-grown [69]. After Zehr et al., [69] we therefore excluded records from all but adult animals (defined by [69] as ≥twice the minimum dam age of reproduction for lemurs housed at the Duke Lemur Center; see Appendix A Table A1 for these). Wild fat-tailed dwarf lemurs, *Cheirogaleus medius*, and grey mouse lemurs undergo seasonal hibernation or torpor, and naturally undergo programmed fattening (sensu [71]) prior to this [72,73], and captive animals likewise demonstrate a similar pattern of annual weight fluctuations [74]. For these two species, most of their representatives’ weights were taken immediately before their inactive periods (i.e., when they were at their heaviest). Because their relative body masses would be biased upwards, we excluded values from these two species from analyses. After these exclusions we were left with records, including some on living conditions, for 691 animals from 20 species. Our last exclusion was to remove species represented by <5 animals (after [75,76]), resulting in a final dataset of 675 animals from 13 species. For each animal in this dataset we calculated the ratio of its most recently taken body mass to its species-typical wild mean, yielding its relative body mass value.

#### 2.2.1. Further Survey Processing for Comparative Analyses

For comparative analyses we calculated species summary statistics from the final individual-level dataset (described in Table 1). As some species did not have ≥5 animals per sex (see Section 2.5.1), we were unable to examine sex differences in comparative analyses (obesity and its related effects do differ between the sexes across primates: [79,80,81]). For our outcome variable, we calculated the median relative body mass of species’ representatives, this yielding species-typical median relative body mass.

Environment and living conditions also contribute to weight gain [13] and, should these covary with our wild ecology predictors, could be confounds [63]. Therefore, using the information on living conditions we collected in our survey, we calculated six husbandry variables (see Table 1) believed *a priori* to likely influence body weight so we could later statistically control for their effects if necessary. For these we calculated an appropriate summary statistic (a median or proportion; see Table 1).

#### 2.2.2. Further Survey Processing for Epidemiological Analyses

Early exploratory analyses [74] revealed that four of our final 13 species had complete cases across all variables enabling epidemiological analyses (n): ring-tailed lemurs (351), black-and-white ruffed lemurs (89), red ruffed lemurs (75), and mongoose lemurs, *Eulemur mongoz* (29). We excluded records from all other species. Because we were interested in possible sex differences, we also excluded any animal of unknown sex (one animal). Maximum recorded captive lifespans of these four species are similar and thus ages are directly comparable (i.e., mongoose lemur = 36.2 years; ring-tailed lemur = 37.3 years; red ruffed lemur = 37.6 years; black-and-white ruffed lemur = 39.4 years [82]). To boost sample sizes, we estimated some dates and corresponding seasons of weighing recorded as “unknown” for the comparative analyses, by assigning the former as the 1st of the month that we received weight records and using this to calculate the corresponding hemisphere-specific season of weighing. For two ring-tailed lemurs with dates of birth but without dates of weighing, we also used these estimated dates to calculate estimated age at weighing. Doing so resulted in them being deemed sub-adult rather than adult as per the comparative analyses, thus excluding them from the epidemiological analyses. Ring-tailed lemurs are adult from 978 days of age (cf. [69]). Being very close to adulthood (929 and 936 days), including these individuals in the comparative analyses where they just contributed to the species median is therefore likely to have minimal impact.

### 2.3. Comparative Analyses Predictor Variable Data Collation

For the 13 species featuring in the comparative analyses, we collated data on six wild ecological predictor variables from published literature to test our hypotheses. See Table 2 for details and rationale of wild ecological predictors and their calculations, and Table 3 for species-typical values.

### 2.4. Predictors for Epidemiological Analyses

Details of husbandry and demographic predictor variables calculated from our survey and their rationale are shown in Table 1.

### 2.5. Statistical Procedures

All analyses were performed in R version 3.6.1 [87].

#### 2.5.1. Comparative Analyses

Prior to hypothesis-testing we made two types of confound check. First, we investigated correlations between wild ecology predictor variables belonging to different hypotheses. Based on these results, we made further checks during hypothesis-testing by including correlated predictors as extra terms, to assess whether they altered interpretation of the focal predictor (in practice they never did, so hypothesis-testing models are reported without them). Our second confound check was to assess relationships between wild ecology predictor variables and the six species-typical husbandry variables shown in Table 1. Correlated husbandry variable(s) were included in final hypothesis-testing models.

To control for species non-independence due to shared ancestry, we used phylogenetic generalised least squares (PGLS) regressions [96,97] for all continuous outcomes [98], and phylogenetic logistic regression models when ground-use was the outcome during between-predictor confound checks (“phylolm” package: [99]). For comparative confound checks, we used a consensus lemur phylogenetic tree (from [100]). Models were only run if data were available for ≥5 species, or ≥5 species per level for categorical variables. Potential outliers were assessed on graphs and PGLS diagnostic plots [98], and results reported with outliers if model assumptions were not violated, and they did not have a studentised phylogenetic residual >±3 (e.g., [101]). Homogeneity of residuals was checked on diagnostic plots, and normality assessed using a Shapiro-Wilk normality test—where necessary transformations were applied to satisfy these. Pagel’s Lambda (λ), a measure of phylogenetic signal [97,102,103], was estimated in PGLS models using maximum likelihood. The phylogenetic logistic regression equivalent of λ is alpha (α), which is reported in relevant models.

To account for phylogenetic uncertainty, all final hypothesis-testing models were performed over a tree block of 1000 alternative lemur phylogenetic trees (from [100]), and summarised as medians and 95% CIs. Results were considered significant at *p* < 0.05 and all *p* values are two-tailed.

#### 2.5.2. Epidemiological Analyses

For epidemiological analyses, we used mixed effects models in ‘lmer4′ [104] and ‘ordinal’ [105]. To account for variance explained by non-independence between animals sharing enclosures, and enclosures within the same zoo, ‘enclosure’ was nested in ‘zoo’ as a random effect. Relative body mass was natural log-transformed to better meet the requirements of linear modelling, as were age and enclosure area because of their skew and hence leverage of high values. During hypothesis-testing, outliers with studentised residuals >±3 (e.g., after: [101]) were removed (four animals) as their inclusion meant that models’ residuals did not pass a Shapiro–Wilks normality test.

Being non-experimental data, the explanatory power of predictor variables (cf. Table 2 in [106]) was assessed by comparing Akaike information criterion (AIC) scores of models with and without a focal predictor [107]. Models with lower AIC scores by ≥2 were judged as improvements [108]. If a focal predictor yielded a better fit to the data than a model without it, the model’s coefficients were examined to interpret the relationship. For categorical predictor terms with >2 factors, we spilt the dataset by that factor and re-ran the analysis to assess the relationship between the focal predictor and relative body mass.

We first checked for relationships between predictors using all data available for each variable. In all cases we used AIC scores to initially assess whether varying intercepts only or intercepts and slopes provided the better fit [109], with the random effects structure mentioned above. Linear mixed models were used when age and enclosure size were analysed as outcomes; generalised linear mixed models (binomial family and logit link function) were used for contraceptive use/neutered status and sex, and for when % fruit (gamma family and inverse link function) and enrichment score (Poisson family and log link function) were analysed as outcomes; and cumulative link mixed models (probit link function) [105] were used when the ordinally ranked enclosure type was an outcome. Relationships between variables were confirmed if the full model with the predictor term was a better fit to the data than null without it [110]. We ran additional models at the univariable stage of hypothesis-testing (below) to assess if correlated predictors affected interpretation of focal predictors, by including them as extra predictor terms (in practice they never did).

To enable model comparisons for hypothesis-testing, next we reduced our dataset to animals with complete data across all variables. We followed a similar model-building approach to KilBride et al. [58], although we evaluated interactions. Initially, we assessed univariable associations between predictors and our outcome using the same linear mixed models and procedure already described (here, varying intercepts and slopes never gave a better fit to the data than varying intercepts only). Predictors found to have a univariable relationship with relative body mass, as determined by AIC, were taken forward to the multivariable model-building stage.

Multivariable model building began with a model including all predictors with a univariable association with relative body mass. We used a forwards multiple regression technique and by making comparisons to the simplest version of this model (i.e., without any interactions), we checked for improvements in model fit by progressively including interactions, stopping when additions did not yield further improvements. This became our minimal adequate (baseline) model. Using this baseline model, we reassessed predictor variables without a univariable association with relative body mass, by sequentially added these into the baseline model, again checking for interactions, to see if they now improved model fit (after: [58,111]). We continued until none of the remaining predictors resulted in improvements, yielding our final minimal adequate model.

## 3. Results

### 3.1. Survey Response Rate and Descriptive Statistics

**Figure 1:**
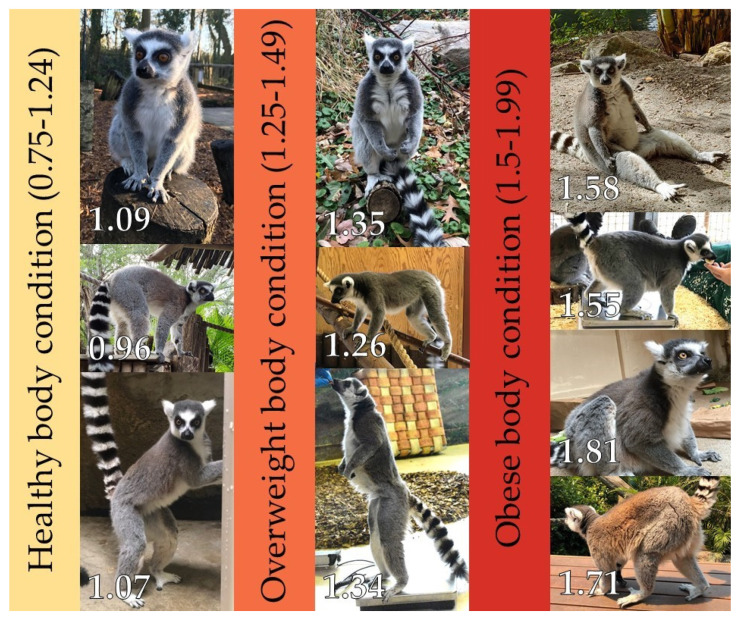
Images of different adult lemurs in one of three body conditions as deemed by their relative body masses: healthy, overweight, or obese. The number at the bottom of each image is that animal’s relative body mass at the time the photograph was taken. Sex of lemurs (credits). **Far left**, **top**: female (Lakeland Wildlife Oasis); **middle**: male (Nancy Nill, Palm Beach Zoo); **bottom**: female (Ashley Ashcraft). **Middle**, **top**: male (Valerie Schultz, Smithsonian’s National Zoo); **middle**: female (Śląski Ogród Zoologiczny); **bottom**: male (Heidi Beal). **Right**, **top**: male (Nancy Nill, Palm Beach Zoo); **second from top**: female (Śląski Ogród Zoologiczny); **second from bottom**: male (Debbie Fenton); **bottom**: male (Rebecca Lambert, Taronga Conservation Society).

Of 359 zoos contacted 135 responded, representing a response rate of 38%. Of the 13 species in our final comparative dataset, their sample sizes and species-typical relative body masses are shown in Table 3. Figure 1 provides a visual depiction of ring-tailed lemurs in different body conditions (note we did not have images of those in underweight and morbidly obese conditions).

**Figure 2:**
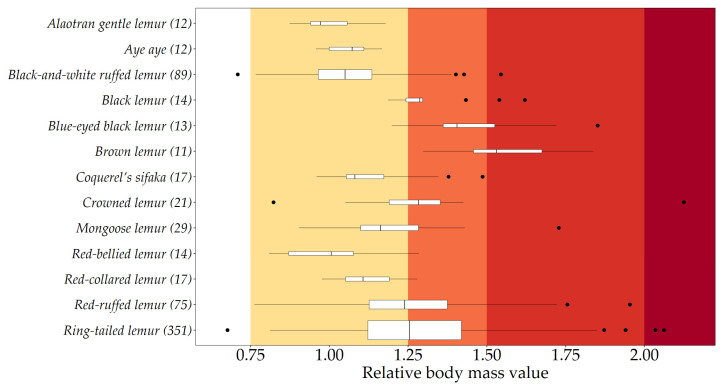
Box-and-whisker plots of relative body mass values of individual adult lemurs, from the 13 species featuring in our comparative analyses. Ring-tailed lemurs, mongoose lemurs, black-and-white ruffed lemurs, and red ruffed lemurs also featured in our epidemiological analyses. Sample sizes are shown in parenthesis next to species’ names, and are also are indicated by the relative width of their respective boxplot. Species’ medians are indicated by vertical lines on the boxes; the extent of the boxes indicate their interquartile ranges; whiskers represent values within 1.5 times the interquartile range; and outliers outside this are depicted as points. The healthy range (0.75–1.25) is shown by yellow shading; overweight by orange (1.25–1.5); obese by red (1.5–2); and morbidly obese (>2) by the darker red.

Boxplots of individuals’ relative body masses are shown in Figure 2, grouped by species. Across our 13 species, 0.30% of animals were underweight (species-typical relative body mass <0.75), 57.63% were healthy (0.75–1.25), 28.30% were overweight (1.25–1.5), 13.33% were obese (1.5–2) and 0.44% were morbidly obese (>2).

Species-typical median relative body mass values (indicated by vertical lines on the boxes of Figure 2) of eight species corresponded with the healthy category: aye ayes, red-collared lemurs, *E. collaris*, mongoose lemurs, red-bellied lemurs, Alaotran gentle lemurs, *Hapalemur alaotrensis*, Coquerel’s sifaka, *Propithecus coquereli*, and black-and-white and red ruffed lemurs. Four species were classed as being typically overweight: crowned lemurs, blue-eyed black lemurs, black lemurs, *E. macaco*, and ring-tailed lemurs. One species, brown lemur, *E. fulvus*, was classed as obese.

### 3.2. Comparative Analyses: Results

**Figure 3:**
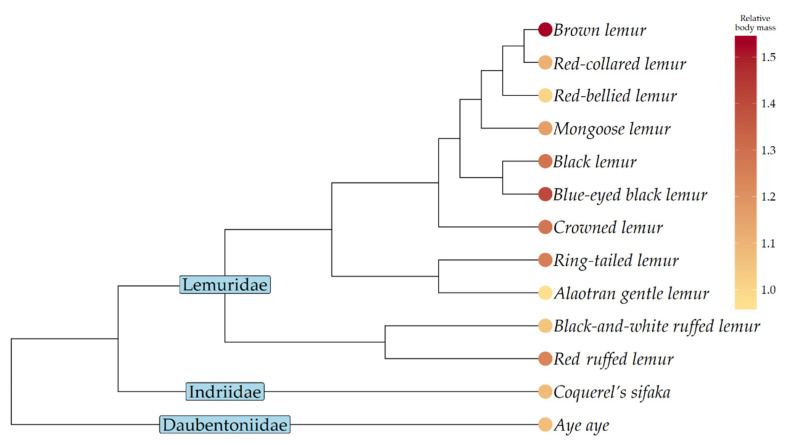
Phylogenetic tree of 13 lemur species in our comparative analyses, with their species-typical median relative body mass values shown as tip-points. The colour of a species’ tip-point represents its species-typical body condition: the healthy range (species-typical relative body mass 0.75–1.25) is shown by yellow shading; overweight by orange (1.25–1.5); obese by red (1.5–2); and morbidly obese (>2) by the darker red.

Figure 3 shows the phylogenetic tree of the 13 species featured in our comparative analyses, with tip-points coloured according to species-typical median relative body mass values.

#### 3.2.1. Comparative Analyses: Results of Confound Checks

All between-predictor confound checks are shown in Appendix A Table A2. Two sets of between-predictor check models were significant. Species that spend more time on the ground live in ranges with low annual rainfall (*t*_6_ = −3.64, R^2^ = 0.69, λ = 0.51, *p* = 0.01), and vice versa (*t*_6_ = −9.31, R^2^ = 0.94, λ = 0, *p* < 0.001). Species more heavily predated also live in ranges with low annual rainfall (*t*_11_ = −3.16, R^2^ = 0.48, λ = 0.77, *p* = 0.01). Results of predictor-husbandry checks are also shown in Appendix A Table A3—no significant relationships between wild ecology predictors and species-typical husbandry were found.

#### 3.2.2. Comparative Analyses: Results of Hypothesis-Testing

**Figure 4:**
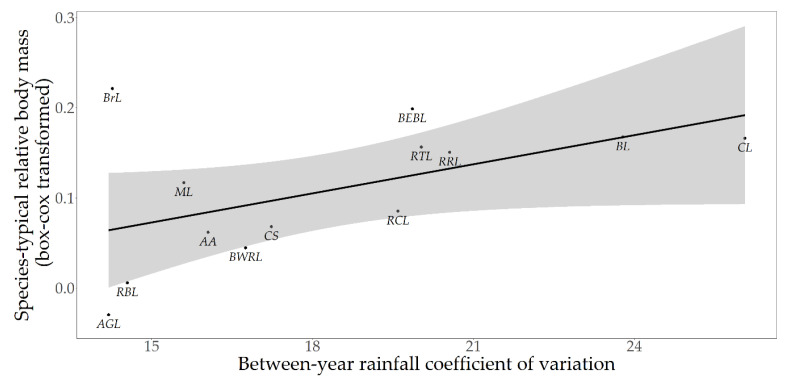
Relationship between between-year rainfall coefficient of variation and species-typical median relative body mass across 13 lemur species (*t*_11_ = 2.04, R^2^ = 0.27, λ < 0.001, *p* = 0.07). The shaded area shows the 95% confidence region. *Labels*: AGL: Alaotran gentle lemur; AA: aye aye; BEBL: blue-eyed black lemur; BL: black lemur; BrL: brown lemur; BWRL: black-and-white ruffed lemur; CL: crowned lemur; RCL: red-collared lemur; RCL: Coquerel’s sifaka; ML: mongoose lemur; RBL: red-bellied lemur; RRL: red ruffed lemur; RTL: ring-tailed lemur.

No aspect of wild ecology significantly predicted species-typical median relative body mass (Table 4); however, we did uncover one trend. Species that experience large between-year variation in rainfall, and thus greater food resource unpredictability, tend to have larger species-typical relative body masses (Figure 4; as the 95% CI values are identical [see Table 4], here we report median values for each parameter: *t*_11_ = 2.04, R^2^ = 0.27, λ < 0.01, *p* = 0.07). To illustrate, black lemurs, crowned lemurs, and ring-tailed lemurs are examples of species that experience relatively large between-year variation in rainfall and, correspondingly, whose representatives are typically overweight in captivity.

### 3.3. Epidemiological Analyses: Results

#### 3.3.1. Epidemiological Analyses: Between-Predictor Checks Results

Results of predictor-predictor checks featuring the four best-sampled species included in the comparative analyses are shown and described in Appendix A Table A4.

#### 3.3.2. Epidemiological Analyses: Univariable Results

The reduced (complete cases) epidemiological dataset contained data for 256 animals. Details of predictors with a univariable association with relative body mass are shown in Appendix A Table A5. In sum, models including species, sex, and age as predictors were found to individually yield better fits to the data than the null model. Including correlated predictors (Section 2.5.2 and Section 3.3.1) did not change the interpretation of focal predictors in relevant models.

#### 3.3.3. Epidemiological Analyses: Multivariable Results

The baseline model with the three predictors found to have a univariable association with relative body mass, is shown in Appendix A Table A6. Species × sex was a better fit to the data than a simpler model without it (AIC = −211.93 v −216.09). Male mongoose lemurs have smaller mean relative body masses, corresponding with healthy, than females, which are typically overweight (*t*_6_ = −2.76, *p* = 0.03); whereas male ring-tailed lemurs have larger mean values than females, but both sexes are typically overweight (*t*_127_ = 3.15, *p* < 0.01). Species and age both had main effects. Black-and-white ruffed lemurs had smaller mean relative body mass values than ring-tailed lemurs (*t*_146_ = −5.11, *p* < 0.001) and red ruffed lemurs (*t*_4_ = −3.94, *p* = 0.02), and relative body mass increased with age (*t*_157_ = 2.09, *p* = 0.04).

**Figure 5:**
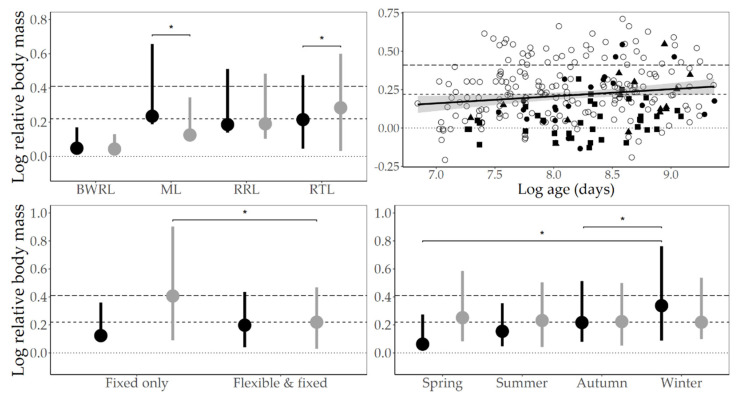
Relationships between relative body mass and species × sex, age, climbing structure provision × sex, and season × sex. Dotted horizontal line indicates the point at which relative body mass = 1 (i.e., the captive animal is the same weight as its species-typical wild mean; 0 on the log-scale); the short dashed line indicates the overweight threshold (≥1.25, or 0.22 on the log-scale); and the long dashed line indicates the obese threshold (≥1.5, or 0.41 on the log-scale). On the three plots with error bars, points indicate the mean relative body mass value, and the whiskers the upper and lower 95% confidence intervals; differences between groups are indicated with asterisks; and females are indicated by black, males by grey. **Top Left**: BWRL: black-and-white ruffed lemur. ML: mongoose lemur. RRL: red ruffed lemur. RTL: ring-tailed lemur. Male mongoose lemurs have smaller relative body masses than females (*t*_6_ = −2.76, *p* = 0.03), whereas male ring-tailed lemurs have larger values than females (*t*_127_ = 3.15, *p* < 0.01). **Top Right**: Relative body mass increases with age (*t*_157_ = 2.09, *p* = 0.04) across four lemur species (both log-transformed): black-and-white ruffed lemur = filled square; mongoose lemur = filled triangle; red ruffed lemur = filled circle; ring-tailed lemur = unfilled circle. The shaded area shows the 95% confidence region. **Bottom Left**: Males housed in enclosures featuring fixed climbing structures only (versus flexible and fixed structures) have larger relative body mass values (*t*_157_ = 3.04, *p* < 0.01). **Bottom Right**: Females weighed in the winter had larger relative body masses than those weighed in spring (*t*_178_ = 3.30, *p* < 0.01) and summer (*t*_25_ = 3.70, *p* < 0.01).

Sequentially adding predictors without a univariable relationship with the outcome into the baseline model yielded the following (see Table 5). Males in enclosures featuring only fixed climbing structures were on average obese, whereas those with flexible and fixed structures were typically overweight (*t*_157_ = 3.04, *p* < 0.01). Females weighed in the winter had larger mean relative body masses, and were normally overweight, than those weighed in spring (*t*_178_ = 3.30, *p* < 0.01) and summer (*t*_25_ = 3.70, *p* < 0.01) which fell within the healthy range. See Figure 5 for visual representations of the multivariable results.

## 4. Discussion

Our comparative study found no support for our hypotheses relating susceptibility to captive weight gain to arboreality, predation risk, or low productivity. However, given our sample sizes were small, likely reducing statistical power (*n* < 20; cf. [112]), we discuss the trend consistent with one hypothesis. Lemurs for which native ranges have large between-year variation in annual rainfall, and who are thus assumed to be adapted to greater food resource unpredictability, tended to have larger relative body masses. They may therefore have “thrifty” adaptations that represent an ecological risk factor for susceptibly to captive weight gain, pre-disposing such species to obesity and its related health problems.

Our epidemiological analyses revealed four demographic and one environmental risk factor for large relative body masses across four species. We confirmed species differences in average relative body masses; found relative body mass to increase with age; and that for two species there was a sex effect. Climbing structure provision influenced relative body masses across males of all species. Males whose climbing structures were fixed had larger relative body masses than those housed with some flexible climbing structures too. Additionally, for females there was a seasonal effect. Next, we discuss findings from each study in turn. Based on these, we then make recommendations for zoos managing these species, describe limitations of our study, and detail ideas for future research.

### 4.1. Comparative Study

Regarding species differences our results show some agreement with two previous multi-species studies ([18]; note we used Terranova and Coffman’s [19] body mass values to calculate the typical body condition of their lemurs ourselves), and our epidemiological analyses likewise confirmed species differences (Table 5). Thus, of species common to all three studies, red-bellied lemurs and black-and-white ruffed lemurs emerged as usually healthy, whereas blue-eyed black lemurs and black lemurs are typically overweight. Mongoose lemurs provide the one instance of disagreement, being typically healthy in our study but overweight in the previous two. This between-study difference might reflect differences in data sources and measurement methods, or recent improvements in captive diet and husbandry for this species (e.g., based on recommendations in: [38]).

How might “thrifty” adaptations to unpredictable wild food resources potentially increase susceptibility to captive weight gain in lemurs? Lemurs have various attributes proposed to facilitate survival in their native, and unpredictable, environment. These include increased resting, food-switching (e.g., from fruit to leaves and flowers), reduced basal metabolic rates and hibernation/torpor during lean periods, as well as seasonal breeding and weaning synchrony [25]. Therefore, lemurs are likely adapted to take advantage of plentiful food when available (cf. [25]) as, presumably, is the case in captive environments. Possibly, if food is delivered in two or three bouts (meals) rather than encountered slowly over the course of a day as per wild food, this might promote binge-eating. If captive food also tends to be energy-rich (cf. [38]) then our result might simply reflect a constant state of positive energy imbalance and subsequent weight gain (sensu [13]).

As unpredictability is key to the potential ecological risk factor identified here, paradoxically, for some species perhaps food unpredictability is being inadvertently signalled by captive feeding regimes, leading to animals going into “thrifty” mode (e.g., increased resting, reduced basal metabolic rate: [25]) ultimately resulting in weight gain. Food unpredictability, rather than greater access to energy-rich food *per se*, is proposed to help explain why people from lower socioeconomic positions in wealthy (but not poorer) countries are at most risk of being overweight or obese [113,114]. For these people, it is the combination of food being sometimes perceived as unpredictable and, when accessible, being energy-rich that might explain this effect [114]. A similar scenario could be relevant for some captive lemurs too. Even if regularly provisioned daily, food provided in a small number of discrete meals rather than being available *ad libitum* might signal unpredictability. This may be especially so for animals in which eating just two or three times per day is likely at odds—a mismatch (cf. [115,116])—with their foraging and eating behaviour during times of plenty in the wild. In humans, attempts to lose weight via episodes of restrictive dieting are usually counterproductive, ultimately resulting in weight gain because such dieting likely cues food insecurity, triggering fat-storage mechanisms when food becomes available [114,117,118,119,120]. For some captive lemurs, then, “thrifty” adaptations combined with being provisioned with energy-rich food (cf. [38,39]) in a small number of daily bouts could lead to weight gain.

### 4.2. Epidemiological Study

We found a species × sex effect in our epidemiological analyses. Because sex-specific wild means were not available for every species in our comparative analyses (see [66]), we used species means for relative body mass calculations. However, the sexes of some species do differ in size, and while these differences have not been found to be statistically significant [18] this could explain our result. Another, more speculative explanation relates to dominance hierarchy. In other taxa, subordinate individuals maintain higher fat reserves than dominants, under scenarios of relatively low energy cost and high food availability (e.g., [121]) like, presumably, captivity. Under such conditions, the trade-off between the risks of death from starvation and death from predation [122] differ according to an animal’s place in the hierarchy [123,124]. Because they do not have feeding priority, subordinates carry greater fat stores as their starvation risk outweighs the cost of carrying extra weight, e.g., increased risk of death by predation [49,123]. Logically, as most lemurs, including our four epidemiological study species, are female-dominant [25,125,126,127] we might expect captive males to be the heavier sex, as we found here for ring-tailed lemurs. But, then, why did we find the *opposite* for mongoose lemurs? A key assumption of the scenario described above, is that subordinate animals should only carry greater fat stores under specific conditions. Under other scenarios, like high starvation risk, dominants are expected to carry greater fat stores [124]. Potentially then, if species differ in their perception of starvation or predation risk, or if food presentation and/or quality is signalled as being unpredictable, one would expect dominant animals, i.e., females, to carry greater fat stores, especially if they get first choice of likely energy-rich captive food (sensu [123,124,128]).

We also revealed an environmental risk factor for large relative body masses in males: being housed with fixed climbing structures only (as opposed to having some flexible structures too). Opportunities for regular exercise/activity may affect weight via an animal’s energy balance, and one can reasonably assume climbing fixed structures requires less physical effort, and therefore less energy expenditure, than does climbing flexible structures (sensu [13]). Exercise-induced weight loss is reported to vary between the sexes in humans: males expend relatively more energy than females, being larger-bodied with larger total daily energy expenditure [129]. A similar effect could explain why male lemurs, and not females, seem more affected by exercise opportunities (but see: [18]). Alternatively, assuming climbing structures are more preferred than the ground, dominant (i.e., female) (cf. [127]) exclusion of subordinates (i.e., males) from the preferred substrate might explain our result, if fixed climbing structures are easier for dominant animals to monopolise. A similar effect might also explain why the lowest ranking squirrel monkeys, *Saimiri sciureus*, in Marriott and Meyers [130] mainly used the ground and feeding areas, instead of suspended logs like their higher-ranking cage mates. Alternatively still, our result could reflect other sex differences in climbing structure use and preference (e.g., female chimpanzees, *Pan troglodytes*, used elevated areas more than males, perhaps to avoid the males’ displays: [131]).

Females’ relative body masses, but not males’, differed seasonally. While known pregnant females were excluded from analyses (see Section 2.2), females with undiagnosed pregnancy could be in our final dataset. Breeding is highly seasonal and often synchronised for wild lemurs (e.g., [12,25]), and captive lemurs also maintain strongly seasonal breeding patterns [132,133]. Thus, the seasonal pattern in relative body mass reported here could reflect pregnancies. Breeding is usually carefully managed in captivity, however. Alternatively, then, our seasonal effect might represent the close association between body condition and (preparation) for reproduction [134,135]. Even if unable to breed, fat stores of females from seasonal breeding species might nevertheless fluctuate in preparation for reproduction (sensu “capital breeding” in: [136]). Our result might therefore indicate key times of the year during which females might be at increased risk of weight gain.

Finally, older animals had larger relative body mass values, in good agreement with other studies of weight-gain and age in non-human primates [137] and humans (inferred using body mass index: [138]). Although very old animals might instead be lighter than those younger, due to effects of chronic illness and other age-related complications (sensu [69]), there was no hint of a non-linear relationship in our data. Examining Figure 5 suggests the older half of our sample are more likely to be overweight, but that the regression line does not cross the obesity threshold. In other words, increasing age does not seem to pose a strong risk for potential obesity. However, note that most of our animals were relatively young: only 14% (37/256) were ≥50% of their species-specific maximum recorded captive lifespan, limiting our ability to extrapolate beyond the current dataset. Therefore, further research is required to determine the health relevance, if any, of the age effect observed in our study.

### 4.3. Recommendations for Zoos

Our results tentatively identify which species—the “thriftier” ones—might benefit most from weight management recommendations (e.g., in: [18,38,39]). Captive diets should be carefully designed to avoid overfeeding calorie-rich diets [38,39], especially for species of concern in Figure 3, e.g., brown lemurs and blue-eyed black lemurs. Limiting/avoiding commercial fruit also seems sensible (also see: [18,38,39]), especially as many wild lemurs readily switch from a primarily fruit-based diet to e.g., leaves and flowers [25]. Factoring appropriate exercise opportunities into enclosures could also help manage weight (also see: [18]), e.g., by providing a mixture of flexible and fixed climbing structures (see Section 4.2); encouraging movement by placing food and other resources such as enrichment at various heights and distances around the enclosure; and necessitating climbing and distances to be covered into enclosures at the initial design stage.

Implicit in our comparative result is that the “thriftiness” enabling some species to succeed in the wild renders the same species prone to captive weight gain. Based on this, we predict which species outside our dataset may likewise be at risk of captive weight gain. Thus, white-fronted brown lemurs, *Eulemur albifrons*, might be at risk because their geographic range is nearby—and so is likely similarly unpredictable—to some of our typically prone species (e.g., crowned lemurs; brown lemurs). Furthermore, one might predict that other taxa with similarly unpredictable wild environments may also be prone to weight gain in captivity.

### 4.4. Limitations

Our sampling method of captive animals is non-random, as we recruited Species360 member zoos. Using survey responses from different people likely introduced noise into our data (see [63]). Being observational rather experimental also means there may be other unmeasured variables not considered here, e.g., calorie intake (sensu [13]), which could affect our outcome and/or be confounded with our predictors. As lemur taxonomy is ever being updated, we cannot rule out potential species (mis)identification especially for *Eulemur* animals [41] within both our captive sample and animals used to calculate species-typical wild means [66]. Our study has unequal representation of families (just one Indriiae and no Cheirogaleidae representatives, the latter for reasons given in Section 2.2), although it is likely representative of lemur species common in captivity (cf. [139]). Seasonal variation was likely represented in some species-typical wild mean weights (cf. [66]), but not all (e.g., [140,141]), which might reduce the representativeness of these as ‘gold standards’ for body mass. We also assumed our wild ecology predictor variables accurately reflect evolved adaptations, e.g., that between-year annual rainfall CV indirectly reflects adaptations to wild food resource unpredictability: an assumption that needs testing. Finally, as a minimum of 20 species is recommended for comparative analyses for acceptable power and Type I error rates [112], these analyses are likely underpowered and should be treated cautiously. This limitation is unlikely to be readily solved; further species are unlikely to be taken into captivity and, if they are (e.g., to avoid extinction), will probably be in small numbers. Nonetheless, these limitations aside, the nature of our study yields results with predictive values as indicators of where problems arise from and how they may be addressed.

### 4.5. Future Research

Relative body mass as a proxy for body condition and the accuracy of its obesity threshold now requires validation, e.g., using lifespan (e.g., from [69]) and/or the prevalence of reproductive problems and other comorbidities of obesity (e.g., from records in ZIMS [142] or from [143]; but see Mellor et al. [63]). If the current threshold of ≥1.5 does correspond with obesity, then such animals should have reduced lifespan and higher prevalence of comorbidities. Alternatively, it might be that “thrifty” species are able to carry relatively greater fat-stores safely, without determinant to health—indeed, this would be adaptive, for wild animals at least. If this were the case, then using obesity thresholds from other taxa (20–25% over the ideal in dogs, cats and humans: [7,13]) would be inappropriate, and even Taylor et al. [18]’s ≥ 50% of species-typical wild mean might prove inaccurate.

Future research might also examine relationships between the potential ecological risk factor identified here, weight gain and the captive feeding environment. Are animals from “thrifty” species simply easier to overfeed? Do some animals binge-eat? Or is food unpredictability accidentally being signalled by food provision schemes (see Section 4.1)? Lastly, a future comparative study might examine whether dominance hierarchy effects do promote fat storage in subordinate individuals, and whether this interacts with the potential ecological risk factor uncovered here (i.e., to assess whether “thriftiness” is more advantageous for subordinates than dominants). If so, one would predict the most despotic species should have the greatest weight difference between subordinates and dominants. This would have management implications; for example, in such species, providing food in multiple sites so it cannot be readily monopolised by dominant individuals.

Finally, the cross-species approach used here clearly highlights species whose typical captive body conditions are concerning, because they deviate the most from their wild norms. Brown lemurs (but see Section 4.4), blue-eyed black lemurs, crowned lemurs, black lemurs, and ring-tailed lemurs (see Figure 3) might therefore be priority species for research effort.

## 5. Conclusions

By using two complementary methods to address our research question, we uncovered one potential ecological, four demographic, and one environmental risk factor for susceptibility to weight gain in captive lemurs. Using phylogenetic comparative methods, we found species that experience large between-year variation in annual rainfall, and thus greater food resource unpredictability, tended to have larger species-typical relative body masses. Using an epidemiological approach, we also report that for some species the sexes were unequally affected: female mongoose lemurs were heavier than males, but male ring-tailed lemurs were heavier than females. Relative body mass also increases with age, though it is unclear if increasing age *per se* is a risk factor for being overweight or obese. Climbing structure provision also affected males: those housed with only fixed climbing structures had larger relative body masses than those housed with a mixture of flexible and fixed structures. Finally, for females there was a seasonal effect, as those weighed in winter had larger relative body masses than those weighed in spring or summer. Based on these results, we were able to make targeted practical recommendations to help address weight concerns. The validation of relative body mass as a proxy for body condition is now required.

## Figures and Tables

**Table 1:** Details of survey data processing. Individual-level variables are those used in the epidemiology analyses (*n* = number of animals with data), and species-level are those used for comparative analyses (*n* = number of species with data). Note that a subset of the total available data, from the four best-sampled species, was used for epidemiological analyses (see Section 2.2.2).

Definition	Levels or Type	Rationale	Individual-Level Variable (*n*)	Species-Level Variable (*n*)	Comments Regarding Species-Level Variable
***Outcome***					
For adults only, the ratio of the most recent mass recorded (grams) to its species-typical wild mean (grams)	Continuous	See Section 2.1	Relative body mass (544)	Median relative body mass (13)	Median across individuals
***Husbandry***					
Type of climbing structures within the enclosure(s) *Fixed:* climbing structures that are rigid and fixed into place, e.g., platforms, bolted down logs *Flexible:* climbing structures that are unstable and flexible, e.g., ropes, branches on a living tree	Fixed only Flexible and fixed ^1^	We assumed fixed climbing structures require less physical effort to climb, and thus might contribute to large body masses, than flexible structures (sensu [13])	Climbing structures (544)	Proportion with some flexible climbing structures ^2^ (13)	Proportion across enclosures
Contraceptive use/neuter status	Yes No Unknown	Contraceptive usage is associated with weight gain in primates [19,62] and castration can cause obesity in other taxa [14]	Contraceptive use/neuter status (excluding “Unknown”s) (491)	Proportion given contraception/neutered (12)	Proportion calculated across animals of known contraceptive status
Total area (m^2^) of the enclosure ^3^	Continuous	Used to infer quantity of exercise space, assuming that less exercise, and increased risk of weight gain, occurs in smaller enclosures (sensu [13])	Enclosure area (469)	Median enclosure area (13)	Median across enclosures
Type of enclosure the animal is housed in. *Note:* Animals with access to indoor and outdoor enclosures for all or part of the year, or any combination of these were scored as ‘indoor and outdoor’	*Ordinally ranked:* Indoor only Indoor and outdoor Outdoor only	Levels were ranked according to an assumed increasing need for thermoregulation, and therefore energy expenditure (sensu [61]) indoor only < indoor and outdoor < outdoor only	Enclosure type (544)	Proportion of enclosures which are indoors	Proportion calculated across enclosures
Types of enrichment scored according to their provision: daily (5), two/three times per week (4), weekly (3), monthly (2), less than monthly (1), never (0). Summed across for a maximum total score of 25 *Enrichment types:* Foraging-related, manipulable, olfactory, audio/visual, and training	Counts	We assumed a less enriched environment represents fewer activity opportunities, which could contribute to weight gain (sensu [13])	Enrichment score (544)	Median enrichment score (13)	Median across enclosures
Proportion of the diet fed that is fruit	Continuous	Commercial, domesticated fruit are more energy-rich than the fruits wild lemurs consume, and if fed in large amounts could contribute to weight gain [38]	Proportion of the diet that is fruit (529)	Median proportion of fruit in the diet (13)	Median across enclosures
***Demographics (epidemiological analyses only)***					
Age at weighing (days)	Continuous	Older animals are at higher risk of weight gain in other primates [5]	Age (365)	-	-
Season of weighing	Spring Summer Autumn Winter	In the wild, some species display seasonal variation in body masses, e.g., [77,78]	Season of weighing (544)	-	-
Sex	Female Male	Risk of obesity and its related health problems varies between the sexes across primates [79,80,81]	Sex (544)	-	-
Species name	Ring-tailed lemur Black-and-white ruffed lemur Red ruffed lemur Mongoose lemur	Species differ in their susceptibility to weight gain in captivity [18,19]	Species name (544)	-	-

^1^ Only one enclosure featured flexible only climbing structures, so this was pooled into the ‘Flexible and fixed’ level for analysis. ^2^ For animals with both indoor and outdoor enclosures, if one had flexible climbing structures but not the other they were recorded as ‘flexible’. ^3^ Summed across indoor and outdoor enclosures.

**Table 2:** Table describing predictor variables used to test our comparative hypotheses, their rationale, calculation, and predicted relationship with species-typical median relative body mass. *n* = number of species with data.

Hypothesis: Susceptibility to Captive Weight Gain Relates to…	Predictor Variable (*n*)	Rationale	Predicted Relationship with Species-Typical Relative Body Mass
(i) low productivity	Annual rainfall (13)	Annual rainfall is commonly used in the primate literature to infer primary productivity [28,29,30], because it directly affects plant growth [26,27] and thus food availability. We extracted the mean annual rainfall within each species’ native geographic range (from [83]) for years 1970–2000 (from [84]) at 1 km^2^ spatial resolution using QGIS (version: Maderia 3.4; QGIS Development [85]), taking a median across range fragments for species with a fragmented range ^a^.	Negative
(ii) unpredictable food resources	Between-year annual rainfall coefficient of variation (13)	We assumed that greater between-year variation in rainfall represents greater unpredictability in primary productivity and, thus, food resource availability (cf. [26,27]). Using 0.5 × 0.5 gridded values, within each species’ geographic range we calculated the mean total rainfall for every month between 1901–2016 (CRU version 4.01 [86]) in R (version 3.6.1 [87]) using the packages “maptools” [88], “raster” [89] and “rgdal” [90]. By summing a given year’s monthly means we calculated annual total rainfall values, then calculated the coefficient of variation of these $CV\;=\;\frac{\sigma }{\mu }\times 100$ Where σ is the standard deviation of annual total rainfall values, and μ is the mean of these values.	Positive
(iii) natural arboreality	Ground use, common v rare (11)	From descriptive terms in published literature, we categorised species’ ground use as “common” (e.g., often, sometimes, regularly forages and/or nests on the ground) or “rare” (e.g., rarely, almost never). Sources were: Mittermeier et al. [41], Garbutt [43], Macdonald [91]; and field studies of native, free-living wild-born lemur populations identified during systematic literature searches in Web of Science and British Library’s EThOS Thesis Repository [92], using species’ scientific and common names as terms. Using a species % scans at ground level (below), we also arbitrarily classed species spending ≥10% scans at ground-level as ‘common’ ^b^.	Common > rare
	Ground time, % scans (8)	We used data from field studies identified in our systematic literature searches to calculate time spent at ground-level (% scans). For species with values from >1 study, we calculated the median value across studies.	Positive
	Relative tail length (13)	The proportion of a species’ total length (head to tail tip) that is the tail (from [41,43]). Larger values correspond to relatively longer tails: an adaptation to arboreality across primates [93,94,95].	Negative
(iv) predation risk	Predation score, 0–3 (13)	Data on direct predation pressure are rare, but broad taxonomic descriptions of predators (e.g., “raptors”) are described [41,43]. We assumed that being predated by an increasing number of different classes of species (i.e., mammals, birds, reptiles) likely represents increasing evolutionary investment in different predator-avoidance strategies by lemurs. We scored the maximum of 1 each for reported mammalian, avian, and/or reptilian predation on a species, and summed across these for a maximum possible score of 3 (from [41,43]).	Negative

^a^ Note that while we could have also calculated annual rainfall values using data from CRU version 4.01 [86], our source here [84] provided these values at a finer, and thus preferred, scale. ^b^ Descriptive terms and behavioural data showed good agreement in all but one case. As the study providing behavioural data of crowned lemurs’, *Eulemur coronatus*, ground-use (1% of scans) was only 4 months long, we judged the field-guides to be more reliable and categorised its ground-used as ‘common’.

**Table 3:** Details of species-typical variables used during comparative analyses. Acronyms are explained at the foot of the table. Species-typical outcome and husbandry data processing and calculations are described in Section 2.2.1 and Table 1. Justification and calculation of species-typical wild ecology predictors are detailed in Table 2. Dashes indicate data were not available for that species.

Species Name	Common Name	Species-Typical Outcome			Species-Typical Wild Ecology						Species-Typical Husbandry					
		RBM	n	SD	AR	ARCV	GU	GT	RTL	PS	MEA	MEE	MPF	PFC	PGC	PHI
*Daubentonia madagascariensis*	Aye aye	1.07	12	0.07	2472.25	16.05	Common	-	0.59	0	26.13	14	0.13	0.9	0	1
*Eulemur collaris*	Red-collared lemur	1.11	17	0.09	1445.59	19.59	Rare	2.5	0.57	2	38.05	18	0.29	1	0.15	0.20
*Eulemur coronatus*	Crowned lemur	1.28	21	0.24	1438.58	26.0	Common	1	0.56	2	72	15	0.24	1	0	0.17
*Eulemur flavifrons*	Blue-eyed black lemur	1.41	13	0.19	1706.97	19.86	Rare	0.6	0.61	2	49.10	14	0.29	0.89	0	0
*Eulemur fulvus*	Brown lemur	1.53	11	0.16	1482.72	14.27	-	-	0.46	2	12.95	18	0.25	0.86	0.45	0.14
*Eulemur macaco*	Black lemur	1.29	14	0.13	1961.93	23.78	Rare	0.58	0.58	2	72.59	14.5	0.05	1	0.5	0.17
*Eulemur mongoz*	Mongoose lemur	1.16	29	0.17	1481.21	15.60	Rare	1	0.59	2	36	18	0.33	1	0.14	0.24
*Eulemur rubriventer*	Red-bellied lemur	1.01	14	0.15	1718.98	14.55	Rare	-	0.56	2	102.25	13.5	0.29	0.83	0	0
*Hapalemur alaotrensis*	Alaotran gentle lemur	0.97	12	0.09	1151.84	14.20	Common	-	0.50	2	20	17	0	1	0.08	0
*Lemur catta*	Ring-tailed lemur	1.25	351	0.24	701.21	20.03	Common	27	0.58	3	142.91	14	0.27	0.90	0.33	0.08
*Propithecus coquereli*	Coquerel’s sifaka	1.08	17	0.14	1527.47	17.23	Common	-	0.54	0	14,188.39	14	0.01	1	-	0
*Varecia rubra*	Red ruffed lemur	1.24	75	0.22	2873.58	20.56	Rare	0.01	0.57	0	227.60	14	0.40	0.93	0.19	0.10
*Varecia variegata*	Black-and white ruffed lemur	1.05	89	0.15	1869.95	16.75	Rare	1.1	0.54	0	144.79	14	0.29	0.97	0.12	0.05

Acronyms: *Outcome variable:* RBM: species-typical median relative body mass; n: number of individuals with outcome data; SD: standard deviation of species-typical median relative body mass. *Species-typical wild ecology predictor variables:* AR: annual rainfall (mm); ARCV: between-year rainfall coefficient of variance; GU: ground use (common v rare); GT: ground time (% scans); RTL: relative tail length (proportion of tail length to whole body length); PS: predation score (0–3). *Species-typical husbandry variables:* MEA: median enclosure area; MEE: median enrichment score; MPF: median proportion of the captive diet that is fruit; PFC: proportion of enclosures with flexible climbing materials; PGC: proportion of captive animals known to be given contraception/neutered. PHI: proportion of enclosures which were indoors.

**Table 4:** Results of hypothesis-testing model results performed over a tree block of 1000 alternative Lemuriform phylogenetic trees. Because values for upper and lower 95% confidence intervals were identical to medians in all cases, here we provide median values for each parameter. CV = coefficient of variation. Results were considered significant at *p* < 0.05, and trends (*p* < 0.10) are italicised. All *p* values are two-tailed.

Hypothesis	Wild Ecology Predictor	Model Output [95% CIs]
(i) low productivity	Total annual rainfall (mm)	*t*_11_ = −0.10, R^2^ < 0.01, λ < 0.01, *p* = 0.92
(ii) unpredictability	Between-year rainfall CV	*t*_11_*= 2.04, R^2^ = 0.27*, *λ < 0.01*, *p = 0.07*
(iii) arboreality	Ground use (some v rare)	*t*_10_ = −0.59, R^2^ = 0.03, λ < 0.01, *p* = 0.57
	Ground time (% scans)	*t*_6_ = −0.31, R^2^ = 0.02, λ < 0.01, *p* = 0.76
	Relative tail length *	*t*_11_ = −0.15, R^2^ < 0.01, λ < 0.01, *p* = 0.89
(iv) predation risk	Predation score (0–3)	*t*_11_ = 1.18, R^2^ = 0.11, λ < 0.01, *p* = 0.26

* This is the proportion of the length of the tail compared to the entire length of the animal, from head to tail tip.

**Table 5:** Final model of predictors that explain relative body mass. Random effects were ‘enclosure’ nested in ‘zoo’. For each predictor, varying intercepts but common slopes were found to be the best fit to the data. n = number of animals. AIC ^−216.08^ = AIC value of the baseline model (the first four predictors are the baseline model itself, hence they do not have AIC values). Coef. = coefficient. SE = standard error. df = degrees of freedom. 95% CI^LL^ = lower 95% confidence interval of the coefficient; 95% CI^UL^ = upper 95% confidence interval of the coefficient.

Predictor Details			Comparisons to Baseline Model	Model Coefficients						
Predictor Variable	Levels	*n*	AIC ^−216.08^	Coef.	95% CI^LL^	95% CI^UL^	SE	df	*t*	*p*
Species	Black-and-white ruffed lemur	36	-	−0.22	−0.37	−0.08	0.08	157	−2.94	<0.01
	Red ruffed lemur	22		−0.10	−0.25	0.06	0.08	157	−1.20	0.23
	Ring-tailed lemur	183		−0.06	−0.19	0.06	0.07	157	−0.92	0.36
	Mongoose lemur (ref)	15		-	-	-	-	-	-	-
Sex	Male	146	-	−0.17	−0.32	−0.01	0.08	157	−2.06	0.04
	Female (ref)	110		-	-	-	-	-	-	-
Age	Days	256	-	0.04	<0.01	0.07	0.02	157	2.09	0.04
Species × Sex Sex ref: female	Male: Black-and-white ruffed lemur	22	-	0.16	0.00	0.32	0.08	157	1.86	0.07
	Male: Red ruffed lemur	13	-	0.13	−0.04	0.30	0.09	157	1.43	0.15
	Male: Ring-tailed lemur	103		0.21	0.07	0.35	0.07	157	2.78	0.01
	Male: Mongoose lemur (ref)	8		-	-	-	-	-	-	-
Climbing structures	Fixed	19	−214.66	−0.93	−0.24	0.06	0.08	27	−1.15	0.26
	Flexible and fixed (ref)	239		-	-	-	-	-	-	-
Season	Spring	37	−220.20	−0.11	−0.24	0.01	0.06	157	−1.76	0.08
	Summer	108		−0.04	−0.12	0.05	0.04	157	−0.83	0.41
	Winter	37		0.13	0.02	0.25	0.06	157	2.18	0.03
	Autumn (ref)	74		-	-	-	-	-	-	-
Sex × Climbing structures Sex ref: female	Male: Fixed	9	−221.01	0.24	0.09	0.39	0.08	157	3.04	<0.01
	Male: Flexible and fixed (ref)	137		-	-	-	-	-	-	-
Sex × Season Sex ref: female	Spring	26	−226.70	0.09	−0.05	0.22	0.07	157	1.22	0.23
	Summer	56		0.07	−0.02	0.17	0.05	157	1.46	0.15
	Winter	18		−0.15	−0.29	−0.01	0.07	157	−2.06	0.04
	Autumn (ref)	46		-	-	-	-	-	-	-

## Supplementary Materials

The following are available online at https://www.mdpi.com/2076-2615/10/8/1443/s1, File S1: copy of survey questions used to collect data on captive lemurs’ body masses and corresponding living conditions.

## Appendix A

**Table A1:** Ages from which each species is considered adult (see Section 2.2 for rationale). Values are rounded to the nearest whole number.

Species Name	Common Name	Dam Minimum Age at Conception (Days)	Adult From: (Days) *
*Daubentonia madagascariensis*	Aye aye	1540	3081
*Eulemur collaris*	Red-collared lemur	599	1197
*Eulemur coronatus*	Crowned lemur	624	1248
*Eulemur flavifrons*	Blue-eyed black lemur	580	1161
*Eulemur fulvus*	Brown lemur	507	1015
*Eulemur macaco*	Black lemur	540	1080
*Eulemur mongoz*	Mongoose lemur	650	1299
*Eulemur rubriventer*	Red-bellied lemur	650	1299
*Hapalemur alaotrensis* *(H. griseus)*	Alaotran gentle lemur	558	1117
*Lemur catta*	Ring-tailed lemur	489	978
*Propithecus coquereli*	Coquerel’s sifaka	964	1927
*Varecia rubra*	Red ruffed lemur	610	1219
*Varecia variegata*	Black-and-white ruffed lemur	588	1175

* Twice the minimum dam age at conception (following: [69]). NB: As the Duke Lemur Center only holds one of the black-and-white ruffed lemur sub-species, and the sub-species are considered very similar, we used this (1175 days) as the cut off for both sub-species in our dataset. One species, Alaotran gentle lemur, *Hapalemur alaotrensis,* is not held by the Duke Lemur Center. We therefore used values from its closest relative, *Hapalemur griseus* (1117 days), as indicated in parentheses.

**Table A2:** Results of models investigating potential relationships among predictor variables belonging to different hypotheses. PGLS models were used for continuous outcomes, and phylogenetic logistic regression was used to analyse ground use as an outcome. CV = coefficient of variation. Dashes indicate that the analysis could not be performed (because neither PGLS nor phylogenetic logistic regression models are appropriate for ordinal outcome variables like predation risk). Results in bold were considered significant at *p* < 0.05, and trends (*p* < 0.10) are italicised. All *p* values are two-tailed.

Hypothesis:	(i) Low Productivity	(ii) Unpredictability	(iv) Arboreality			(v) Predation Risk
Outcome: Predictor:	Annual Rainfall (mm)	Between-Year Rainfall CV	Ground Use (Rare v Some)	Ground Time (% Scans)	Relative Tail Length *	Predation Score (0–3)
**Annual rainfall (mm)**		*t*_11_ = 0.25, R^2^ = 0.01, λ = 0, *p* = 0.81	Z = −1.25, α = 0.04, *p* = 0.21	***t*_6_ = −9.31, R^2^ = 0.94,** **λ = 0, *p* < 0.001**	*t*_11_ = 0.34, R^2^ = 0.01, λ = 0, *p* = 0.74	-
**Between-year rainfall CV**	*t*_11_ = 0.40, R^2^ = 0.01, λ = 0.90, *p* = 0.70		Z= 0.04, α = 0.08, *p* = 0.97	*t*_6_ = −0.23, R^2^ = 0.01, λ = 0, *p* = 0.83	*t*_10_ = 1.11, R^2^ = 0.11, λ = 0, *p* = 0.29 ^a^	-
**Ground use** **(rare v some)**	*t*_10_ = −1.66, R^2^ = 0.22, λ = 0.65, *p* = 0.13	*t*_10_ = 0.02, R^2^ < 0.001, λ = 0, *p* = 0.99				-
**Ground time (% scans)**	***t*** **_6_** **= −3.64, R^2^ = 0.69,** **λ** **= 0.51,** ***p*** **= 0.01**	*t*_6_ = −0.23, R^2^ = 0.01, λ = 0, *p* = 0.83				-
**Relative tail length ***	*t*_11_ = 0.29, R^2^ = 0.01, λ = 0.91, *p* = 0.78	*t*_10_ = 1.47, R^2^ = 0.18, λ = 0, *p* = 0.17 ^a^				-
**Predation score (0–3)**	***t*** **_11_** **= −3.16, R^2^ = 0.48,** **λ** **= 0.77,** ***p*** **= 0.01**	*t*_11_ = 0.52, R^2^ = 0.02, λ = 0, *p* = 0.61	Z < 0.01, α = 0.10, *p* = 0.99	*t*_6_ *= 2.19, R* ^2^ *= 0.45,* *λ = 0, p = 0.07*	*t*_11_ = −0.01, R^2^ <0.001, λ = 0, *p* = 0.99	

* This is the length of the tail as a proportion of the entire length of the animal, from head to tail tip. Outlier removed: ^a^ *Eulemur fulvus.*

**Table A3:** Results of PGLS models investigating potential relationships between species-typical husbandry and wild ecology predictor variables. In all models the husbandry variables are outcome variables. Dashes indicate an analysis could not be performed due to too few species per level for ground use (see Section 2.5.1). CV = coefficient of variation. Results in bold were considered significant at *p* < 0.05, and trends (*p* < 0.10) are italicised. All *p* values are two-tailed.

Hypothesis:	Wild Ecology Predictor	Median Enclosure Area (m^2^)	Median Enrichment Score (0–25)	Median Proportion of Diet Fruit	Proportion Given Contraception	Proportion Housed Indoors	Proportion with Flexible Climbing Materials
(i) low productivity	**Total annual rainfall (mm)**	*t*_11_ = 0.23, R^2^ = 0.01, λ < 0.01, *p* = 0.82	*t*_11_ = −1.27, R^2^ = 0.13, λ = 0, *p* = 0.23	*t*_11_ = 0.68, R^2^ = 0.04, λ = 0, *p* = 0.51	*t*_10_ = −0.61, R^2^ = 0.04, λ = 0, *p* = 0.56	*t*_11_ = 0.73, R^2^ = 0.05, λ = 1, *p* = 0.48	*t*_11_ = −0.32, R^2^ = 0.01, λ = 0, *p* = 0.76
(ii) unpredictability	**Between-year rainfall CV**	*t*_11_ = 1.64, R^2^ = 0.20, λ = 1, *p* = 0.13	*t*_11_ < 0.001, R^2^ = 0.08, λ = 0, *p* = 0.36	*t*_11_ = 0.26, R^2^ = 0.01, λ = 0, *p* = 0.80	*t*_10_ = 0.39, R^2^ = 0.02, λ = 0, *p* = 0.70	*t*_11_ = 1.37, R^2^ = 0.15, λ = 1, *p* = 0.20	*t*_11_ = 1.27, R^2^ = 0.13, λ = 0, *p* = 0.23
(iii) arboreality	**Ground use** **(some v rare)**	*t*_10_ = 0.35, R^2^ = 0.01, λ = 1, *p* = 0.73	*t*_10_ = −0.34, R^2^ = 0.01, λ = 0, *p* = 0.74 ^a^	*t_10_ = −2.16,* *R^2^ = 0.32,* *λ = 0,* *p = 0.06*	-	*t*_10_ = 0.28, R^2^ = 0.01, λ = 0.97, *p* = 0.79	*t*_10_ = 0.33, R^2^ = 0.01, λ = 0, *p* = 0.75
	**Ground time** **(% scans)**	*t*_6_ = 0.35, R^2^ = 0.01, λ = 1, *p* = 0.73	*t*_6_ = 0.46, R^2^ = 0.03, λ = 0, *p* = 0.66	*t*_6_ = −1.05, R^2^ = 0.15, λ = 0.06, *p* = 0.34	*t*_6_ = 0.26, R^2^ = 0.01, λ = 0, *p* = 0.81	*t*_6_ = −0.15, R^2^ < 0.01, λ = 0, *p* = 0.88	*t*_6_ = −0.01, R^2^ < 0.01, λ = 0, *p* = 0.99
	**Relative tail length ***	*t*_11_ = −0.46, R^2^ = 0.02, λ = 0, *p* = 0.66	*t*_11_ = −1.64, R^2^ = 0.20, λ = 0, *p* = 0.13	*t*_11_ = 0.28, R^2^ = 0.01, λ = 0, *p* = 0.79	*t*_10_ = −1.27, R^2^ = 0.14, λ = 0, *p* = 0.23	*t*_11_ = 0.38, R^2^ = 0.01, λ = 0.98, *p* = 0.71	*t*_11_ = −0.28, R^2^ = 0.01, λ = 0, *p* = 0.79
(iv) predation risk	**Predation score** **(0–3)**	*t*_11_ = −0.47, R^2^ = 0.02, λ = 0, *p* = 0.66	*t*_11_ = 1.36, R^2^ = 0.15, λ = 0, *p* = 0.20	*t*_11_ = 0.27, R^2^ = 0.01, λ = 0, *p* = 0.80	*t*_10_ = 0.65, R^2^ = 0.04, λ = 0, *p* = 0.53	*t*_11_ = −0.22, R^2^ < 0.01, λ = 0.98, *p* = 0.83	*t*_11_ = −0.26, R^2^ = 0.01, λ = 0, *p* = 0.80

* This is the proportion of the length of the tail compared to the entire length of the animal, from head to tail tip. ^a^ Note that the residuals from this model did not pass a Shapiro-Wilks normality test, despite numerous transformation attempts, so this result should be treated cautiously.

**Table A4:** Results of between-predictor checks for the epidemiological analyses. Note that species and season, being categorical with >2 levels and nominal, were not analysable as outcome variables. In all cases, we first checked if varying intercepts only or intercepts and slopes were the best fit. In practice, varying intercepts and slopes were rarely a better fit than intercepts only, and these are indicated with an asterisk. Model comparisons were then made between the full model including the focal predictor and the null, using AIC (see Section 2.5.2). Associations between variables were confirmed if the full model yielded an improved fit, i.e., a lower AIC score by ≥ 2. The AIC of the null model is reported first. Full models found to be a better fit to the data than the null are shown in bold. For these, we assessed the nature of the relationship between the predictor and the outcome using the model’s coefficients, and these are described in the text immediately below the table.

Outcome: Predictor:	Sex	Age	Contraceptive Use/Neutered	Enclosure Area	Enclosure Type	Climbing Structures	Enrichment Score	% Fruit
**Species** Ref: Mongoose lemur	AIC: 708.84 v 711.28	**AIC: 640.79 v 637.73**	AIC: 423.59 v 425.24	**AIC: −405.81 v −434.91**	**AIC: 94.61 v 86.60**	AIC: 40.63 v 45.88	AIC: 2775.40 v 2780.60	AIC: 3376.50 v 3377.90
**Sex** Ref: Female		AIC: 640.79 v 641.64	AIC: 300.71 v 302.57 *	AIC: −413.46 v −412.53 *	AIC: 94.61 v 96.18	AIC: 40.63 v 46.59	AIC: 2775.40 v 2777.30	AIC: 3376.50 v 3377.90
**Age** (days)	AIC: 484.64 v 485.31		AIC: 296.92 v 298.27	AIC: −190.50 v −191.78	AIC: 48.33 v 50.22	AIC: 22.46 v 24.46	AIC: 1870.70 v 1872.70	AIC: 2468.80 v 2467.60
**Season** Ref: Autumn	**AIC: 708.84 v 699.01**	AIC: 640.79 v 640.31	AIC: 423.59 v 426.35	**AIC: −405.81 v −445.78**	AIC: 94.61 v 99.45	AIC: 40.63 v 46.58	AIC: 2775.40 v 2777.70	AIC: 3376.50 v 3379.90
**Contraceptive use/neutered** Ref: No	**AIC: 557.99 v 551.91 ***	AIC: 531.78 v 532.93		AIC: −451.96 v −452.54	AIC: 94.55 v 96.55	AIC: 40.58 v 42.23	AIC: 2532.60 v 2534.40	AIC: 2800.20 v 2800.40
**Enclosure area** (m^2^)	AIC: 620.58 v 622.55	AIC: 569.46 v 571.32	AIC: 358.18 v 359.13		AIC: 61.79 v 62.44	AIC: 36.79 v 35.71	AIC: 2385.00 v 2386.80	AIC: 2759.60 v 2761.40
**Enclosure type** Ref: Indoor and outdoor	AIC: 708.84 v 710.82	AIC: 640.79 v 642.23	AIC: 423.59 v 424.83	**AIC: −405.81 v −408.53**		AIC: 40.63 v 44.60	**AIC: 2775.40 v 2773.3**	AIC: 3376.50 v 3377.80
**Climbing structures** Ref: Flexible and fixed	AIC: 708.84 v 710.61	AIC: 640.79 v 642.79	AIC: 423.59 v 425.58	**AIC: −405.81 v −408.61**	AIC: 94.61 v 96.60		AIC: 2775.40 v 2777.40	AIC: 3376.50 v 3378.50
**Enrichment score** (0–25)	AIC: 704.60 v 706.06 *	AIC: 640.79 v 642.28	**AIC: 405.16 v 423.59 ***	**AIC: −405.81 v −2832.36**	AIC: 95.22 v 96.96 *	AIC: 40.63 v 42.63		AIC: 3376.50 v 3376.10
**% fruit** (0–100%)	AIC: 690.20 v 690.38	AIC: 620.37 v 620.90	**AIC: 414.34 v 407.11**	AIC: −372.03 v −370.67	AIC: 96.30 v 98.07 *	AIC: 40.62 v 42.35	AIC: 2697.80 v 2696.90	

When sex was the outcome variable, the models including contraceptive use/neutered and season as predictors were better fits to the data than the null. Animals given contraception were less likely to be male (Z = −2.91, *p* < 0.01); and animals weighed in spring are more likely to be male (autumn v: spring: Z = 2.98, *p* < 0.01; summer: Z = −1.06, *p* = 0.29; winter: Z = 0.80, *p* = 0.42).

For the age models, the model with species as a predictor was a better fit to the data than the null. Ring-tailed lemurs were younger than mongoose lemurs (*t*_241_ = −2.34, *p* = 0.02), as were, to a lesser extent, black-and-white ruffed lemurs (*t*_241_ = −1.73, *p* = 0.08; red ruffed v mongoose lemurs: *t*_241_ = −0.65, *p* = 0.52).

For the contraceptive use/neutered models, the models with enrichment score and percentage fruit as predictors were better fits to the data than the null. Animals in more enriched enclosures are more likely to be given contraception/neutered (Z = 3.39, *p* < 0.01); and animals fed less fruit were more likely to be given contraception/neutered (Z = −2.71, *p* = 0.01).

For the enclosure area models, models including species, season, enclosure type, climbing structures, and enrichment score as predictors were all better fits than the null. Compared with mongoose lemurs, red ruffed lemurs are housed in smaller enclosures (mongoose lemur v: red ruffed lemurs: t_315_ = −1.86, *p* = 0.06; ring-tailed lemurs: *t*_315_ = 0.64, *p* = 0.52; black-and-white ruffed lemurs: *t*_315_ = 0.55, *p* = 0.58). Animals weighed in the spring (*t*_315_ = −3.67, *p* < 0.001), summer (*t*_315_ = −3.05, *p* < 0.01), and winter (*t*_315_ = −7.00, *p* < 0.001) had smaller enclosures than those weighed in the autumn. Indoor only enclosures were smaller than indoor and outdoor enclosures (indoor v indoor and outdoor: t_67_ = −2.19, *p* = 0.03; outdoor v indoor and outdoor: *t*_67_ = −1.61, *p* = 0.11). Enclosures featuring fixed climbing structures only were smaller (*t*_68_ = −2.21, *p* = 0.03), and higher enrichment scores were also associated with smaller enclosures (*t*_317_ = −1037.32, *p* < 0.001).

For the enclosure type models, the model including species as a predictor was a better fit than the null. Compared with mongoose lemurs, both black-and-white ruffed and red ruffed lemurs are more often housed outdoors (Z = 3.88, *p* < 0.001; Z = 4.78, *p* < 0.001).

For the enrichment scores models, the model with enclosure type as a predictor was a better fit to the data than the null. Outdoor enclosures have higher enrichment scores compared with indoor and outdoor enclosures (outdoor v indoor and outdoor: Z = 2.43, *p* = 0.01; indoor v indoor and outdoor: Z < 0.01, *p* = 0.99).

Thus, predictors identified here as being related had their possible effects on focal predictors assessed during the univariable stage, by including these correlated terms in the relevant models (see also Section 2.5.2).

**Table A5:** Details of predictor variables found to have a univariable relationship with the epidemiological outcome, relative body mass. Random effects were ‘enclosure’ nested in ‘zoo’. For each predictor varying intercepts, but common slopes, were found to be the best fit to the data. n = number of animals. AIC ^−185.23^ = AIC value of the null model (i.e., without predictor terms). Coef. = coefficient. SE = standard error. df = degrees of freedom.

Predictor Details			Comparisons to Null	Model Coefficients				
Predictor Variable	Levels	*n*	AIC ^−185.23^	Coef.	SE	df	*t*	*p*
Species	Black-and-white ruffed lemur	36	−206.70	−0.17	0.06	169	−2.80	0.01
	Red ruffed lemur	22		−0.05	0.06	169	−0.78	0.44
	Ring-tailed lemur	183		0.03	0.05	169	0.49	0.62
	Mongoose lemur (ref)	15		-	-	-	-	-
Sex	Male	146	−189.18	0.05	0.02	171	2.45	0.02
	Female (ref)	110		-	-	-	-	-
Age	Days	256	−187.89	0.04	0.02	171	2.16	0.03

**Table A6:** Details of baseline model featuring three predictors, and one interaction, all previously identified as having a univariable relationship with relative body mass (Table A5). The species × sex interaction was found to be a better fit to the data than a simpler model without it (AIC = −211.93 v −216.09). Random effects were ‘enclosure’ nested in ‘zoo’. For each predictor varying intercepts but common slopes were found to be the best fit to the data. n = number of animals. Coef. = coefficient. SE = standard error. df = degrees of freedom.

Predictor Details			Model Coefficients				
Predictor Variable	Levels	*n*	Coef.	SE	df	*t*	*p*
Species	Black-and-white ruffed lemur	36	−0.22	0.08	164	−2.91	<0.01
	Red ruffed lemur	22	−0.10	0.08	164	−1.20	0.23
	Ring-tailed lemur	183	−0.05	0.07	164	−0.68	0.50
	Mongoose lemur (ref)	15	-	-	-	-	-
Sex	Male	146	−0.10	0.07	164	−1.47	0.14
	Female (ref)	110	-	-	-	-	-
Age	Days	256	0.05	0.02	164	2.60	0.01
Species × Sex Sex ref: female	Male: Black-and-white ruffed lemur	22	0.14	0.09	164	1.60	0.11
	Male: Red ruffed lemur	13	0.12	0.09	164	1.32	0.19
	Male: Ring-tailed lemur	103	0.17	0.07	164	2.39	0.02
	Male: Mongoose lemur (ref)	8	-	-	-	-	-
