# Supplementary material for: Large Lemurs: Ecological, Demographic and Environmental Risk Factors for Weight Gain in Captivity
Source: Animals (Basel). 2020 Aug 18;10(8):1443. doi: 10.3390/ani10081443 (PMC7460476; doi:10.3390/ani10081443)
Supplement: Supplementary file 1 [file animals-10-01443-s001.pdf]

Supplementary Materials S1: copy of survey questions used to collect data on captive lemurs' body masses and corresponding living conditions

Note that the survey was produced in Google Forms, and contained drop down menus, multiple choice boxes, and tick boxes (depending upon the nature of a given question).

Lemur Survey
Section 1: Basic information
Please note that throughout this survey unless specified we require information for ADULT lemurs only.

- Name:
- Position held:
- Email address (please note that future project reports can only be sent if an email address is provided):
- Name of zoo:

Section 2: Enclosure 1

- Which species of lemur is housed in Enclosure 1? Please choose one: *List option of all species of lemur currently held in captivity.*
- How many adult males are there?
- How many adult females are there?
- How many sub-adults, juveniles and/or infants are there?

Enclosure and enrichment

- Which option best describes this enclosure? *List options of: outdoor and indoor available all year round; outdoor available part of the year, indoor available all year; outdoor only; indoor only; prefer not to say; other*
- Is the outdoor enclosure closed- or open-topped?
- What are the approximate dimensions of the outdoor enclosure? Please give the length x width x height of the enclosure in meters. If the enclosure is open-topped please give the approximate height of the tallest tree/climbing structure:
- Please indicate the types of climbing structures present in the outdoor enclosure. Check all that apply: *List options of: climbing structures that are rigid and fixed into place (e.g. platforms, bolted down logs); climbing structures that are unstable and flexible e.g. ropes, branches on a living tree; none; N/A*
- What are the approximate dimensions of the indoor enclosure? Please give the length x width x height of the enclosure in meters:
- Please indicate the types of climbing structures present in the indoor enclosure. Check all that apply: *List options of: climbing structures that are rigid and fixed into place (e.g. platforms, bolted down logs); climbing structures that are unstable and flexible e.g. ropes, branches on a living tree; none; N/A*
- Which types and how often is environmental enrichment (EE), if any, usually offered in this enclosure? *Following options set out in rows, with columns with options to indicate the frequency for each.* Foraging EE (e.g. Kongs, feeding tubes, feeding puzzles, food placed into boxes); Manipulable EE (e.g. tyres, empty boxes, Christmas trees, toys [without food]); Olfactory EE (e.g. scents placed around the enclosure, essential oils, faeces from an unfamiliar animal); Sound/visual EE (e.g. radio, music or other sounds played, television); Training offered

Other enclosures

- Do you have other lemur enclosures within your collection that you wish to describe here? *Yes/No answer. The participant's answer determines which questions they see next. If 'Yes' then the same set of enclosure questions as above appear (with space for up to 10 enclosures). If 'No' then they go on to the following section*

Section 3: Diet
To save time, if you are able to provide copies of diet sheets for the adult lemurs in your care, indicating if the amounts fed are per adult lemur or for the adults overall, please attach them in an email to: em15953@bristol.ac.uk
Please note that only information for adult lemurs is required.

- Are you able to provide copies of diet sheets for the lemurs in your care? *Yes/No answer. The participant's answer determines which questions they see next. If 'Yes' they go on to the next section on body masses. If 'No' the following dietary questions appear*

Dietary information: Species 1

- Which species is fed this diet? *List option of all species of lemur currently held in captivity.*
- Please describe the typical diet fed to this species in grams per adult lemur per day. Please specify exact types (e.g. '100g apple, 50g lettuce' rather than '150g fruit and vegetables') and, where applicable, brand names:
- How many times per day (including snacks) is this species typically fed?
- Is there another species whose diet you wish to describe here? *Yes/No answer. The participant's answer determines which questions they see next. If 'Yes' the same dietary questions as above appear. If 'No' they go on to the next section on body masses.*

Section 4: Individual information
To save time, if you are able to provide copies of ZIMS Specimen reports for the adult lemurs in your care (including body masses and contraceptive status showing), please attach them in an email to: em15953@bristol.ac.uk
Please note that only information for adult lemurs is required.

- Are you able to provide copies of ZIMS Specimen reports for the adult lemurs in your care? *Yes/No answer. The participant's answer determines which questions they see next. If 'Yes' they go on to the next section on body masses. If 'No' the following dietary questions appear*

Individual information: Species 1

- Which species are you describing here? *List option of all species of lemur currently held in captivity.*
- Are any of these animals currently given contraceptives or neutered (de-sexed)? *Yes/No/Prefer not to say. The participant's answer determines which questions they see next. If 'Yes' they see the first set of questions below, if 'No' they see the second, and if they answer 'Prefer not to say' they see the third.*

Body masses of Species 1 (*these are the 'Yes' questions*)
Body masses of animals who ARE currently given contraceptives / neutered (de-sexed)

- For the adult males of this species, please give the weight, in grams (g), of each individual who IS currently given contraceptives / neutered (de-sexed, and the date (month/year) of weighing):
- For the non-pregnant adult females of this species, please give the weight, in grams (g), of each individual who IS currently given contraceptives / neutered (de-sexed), and the date (month/year) of weighing:

Body masses of animals NOT currently given contraceptives / neutered (de-sexed)

- For the adult males of this species, please give the weight, in grams (g), of each individual NOT currently given contraceptives / neutered (de-sexed), and the date (month/year) of weighing:
- For the non-pregnant adult females of this species, please give the weight, in grams (g), of each individual NOT currently given contraceptives / neutered (de-sexed), and the date (month/year) of weighing:
- For the pregnant adult females of this species, please give the weight, in grams (g), of each individual, and the date (month/year) of weighing:

Body masses of Species 1 (*these are the 'No' questions*)

- For the adult males of this species, please give the weight, in grams (g), of each individual, and the date (month/year) of weighing:
- For the non-pregnant adult females of this species, please give the weight, in grams (g), of each individual, and the date (month/year) of weighing:
- For the pregnant adult females of this species, please give the weight, in grams (g), of each individual, and the date (month/year) of weighing:

Body masses of Species 1 (*these are the 'Prefer not to say' questions*)

- For the adult males of this species, please give the weight, in grams (g), of each individual, and the date (month/year) of weighing:
- For the non-pregnant adult females of this species, please give the weight, in grams (g), of each individual, and the date (month/year) of weighing:
- For the pregnant adult females of this species, please give the weight, in grams (g), of each individual, and the date (month/year) of weighing:
- *At the end of each of these three subsections is this question:*
- Is there another species you wish to describe here? *Yes/No answer. The participant's answer determines which questions they see next. If 'Yes' the same individual information questions appear. If 'No' the following end of survey box appears*

Thank you for participating in this survey! If you are providing copies of diet sheets and/or ZIMS Specimen reports, please email them to: em15953@bristol.ac.uk
